# Genomic insights into the population structure and history of the Irish Travellers

**DOI:** 10.1038/srep42187

**Published:** 2017-02-09

**Authors:** Edmund Gilbert, Shai Carmi, Sean Ennis, James F. Wilson, Gianpiero L. Cavalleri

**Affiliations:** 1 Molecular and Cellular Therapeutics, Royal College of Surgeons in Ireland, St Stephen’s Green, Dublin 2, Ireland; 2 Braun School of Public Health and Community Medicine, The Hebrew University of Jerusalem, Jerusalem, Israel; 3 School of Medicine and Medical Science, University College Dublin, Dublin, Ireland; 4 Centre for Global Health Research, Usher Institute for Population Health Sciences and Informatics, University of Edinburgh, Teviot Place, Edinburgh, Scotland; 5 MRC Human Genetics Unit, Institute of Genetics and Molecular Medicine, University of Edinburgh, Western General Hospital, Crewe Road, Edinburgh, Scotland

## Abstract

The Irish Travellers are a population with a history of nomadism; consanguineous unions are common and they are socially isolated from the surrounding, ‘settled’ Irish people. Low-resolution genetic analysis suggests a common Irish origin between the settled and the Traveller populations. What is not known, however, is the extent of population structure within the Irish Travellers, the time of divergence from the general Irish population, or the extent of autozygosity. Using a sample of 50 Irish Travellers, 143 European Roma, 2232 settled Irish, 2039 British and 6255 European or world-wide individuals, we demonstrate evidence for population substructure within the Irish Traveller population, and estimate a time of divergence before the Great Famine of 1845–1852. We quantify the high levels of autozygosity, which are comparable to levels previously described in Orcadian 1^st^/2^nd^ cousin offspring, and finally show the Irish Traveller population has no particular genetic links to the European Roma. The levels of autozygosity and distinct Irish origins have implications for disease mapping within Ireland, while the population structure and divergence inform on social history.

The Irish Travellers are a community within Ireland, consisting of between 29,000–40,000 individuals, representing 0.6% of the Irish population as a whole^1^. They are traditionally nomadic, moving around rural Ireland and providing seasonal labour, as well as participating in horse-trading and tin-smithing^2^. Since the 1950’s the need for such traditional services has declined^3^, and the population has become increasingly urban, with the majority living within a fixed abode^1^. Despite this change in lifestyle, the Traveller community remains tight-knit but also socially isolated. The population has its own language^4^, known as Shelta, of which Cant and Gammon are dialects.

There is a lack of documentary evidence informing on the history of the Irish Traveller population^5^,^6^. As a result, their origins are a source of considerable debate, with no single origin explanation being widely accepted. It has been suggested that the Irish Travellers are a hybrid population between settled Irish and Romani gypsies, due to the similarities in their nomadic lifestyle. Other, “Irish Origin”, hypothesised sources of the Irish Travellers include; displacement from times of famine (such as between 1740–1741, or the Great Famine of 1845–1852), or displacement from the time of Cromwellian (1649–53) or the Anglo-Norman conquests (1169 to 1240). The Irish Traveller population may even pre-date these events, and represent Celtic or pre-Celtic isolates^4^. These models of ethnogenesis are not necessarily mutually exclusive, and the Irish Traveller population may have multiple sources of origin with a shared culture.

Consanguineous marriages are common within the Irish Traveller community^7^,^8^. Small, isolated and endogamous populations such as the Travellers are also more prone to the effects of genetic drift. The isolation and consanguinity have in turn led to an increased prevalence of recessive diseases^7^,^9^,^10^, with higher incidences of diseases such as transferase-deficient galactosaemia^11^,^12^, and Hurler syndrome^13^ observed in the Traveller population relative to the settled Irish. However, the extent of autozygosity within the population has yet to be quantified; as a result it is unknown how homozygous the population is compared to other, better-studied, isolated European populations.

Previous work into the genetics of the Irish Traveller population has been conducted on datasets of relatively low genetic resolution. A recent study used blood groups to investigate the population history of the Irish Travellers^2^. Multivariate analysis of genotype data across 12 red blood cell loci in 119 Irish Travellers suggested that the population clustered closely with the settled Irish to the exclusion of the Roma. They did, however, appear divergent from the settled Irish. The authors attributed the source of such divergence to genetic drift - but were unable to determine whether any such drift was due to a founder effect, or sustained endogamy. Studies of Mendelian diseases suggest that pathogenic mutations in the settled Irish population are often the same as those observed in the Traveller population such is the case for tranferase-deficient galactosaemia (Q118R in the GALT gene^11^) and Hurlers Syndrome (W402X, in the α-l-iduronidase gene^13^).

Using dense, genome-wide, SNP datasets which provide much greater resolution than genetic systems studied in the Travellers to date, we set out to i) describe the genetic structure within the Traveller population, ii) the relationship between the Irish Travellers and other European populations, iii) estimate the time of divergence between the Travellers and settled Irish, and iv) the levels of autozygosity within the Irish Traveller population.

## Results

### Population Structure of the Irish Travellers

**Figure 1:**
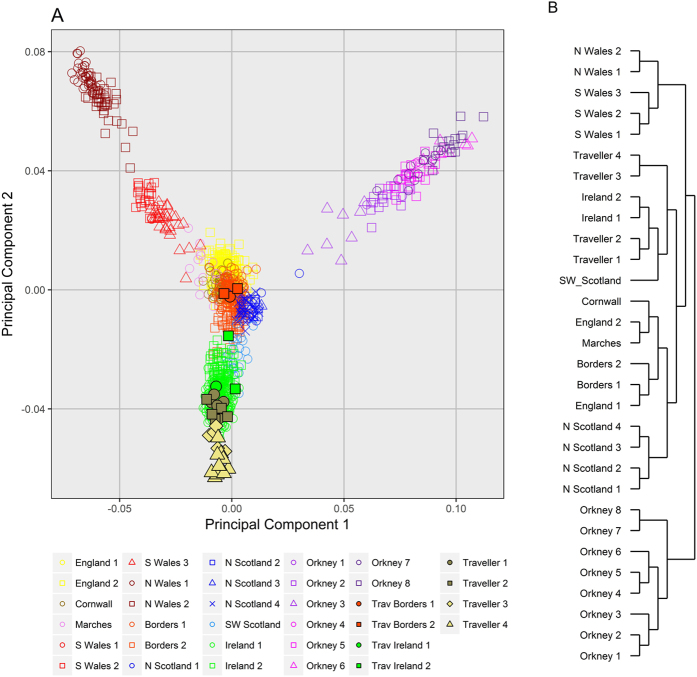
Clustering of 34 Irish Travellers, 300 Settled Irish, and 828 British by fineStructure. (**A**) The first and second components of principal component analysis of the haplotype-based co-ancestry matrix produced by fineStructure analysis. Individual clusters are indicated by colour and shape. Individual Irish Travellers are indicated with black bordered shapes, with cluster shown in Legend. (**B**) The full fineStructure tree with the highest posterior probability, with cluster size and name, and broad branches shown.

In order to investigate the genetic relationship between the Irish Travellers and neighbouring populations we performed fineStructure analysis on Irish Travellers, settled Irish from a subset of the Trinity Student dataset^14^, and British from a subset of the POBI dataset^15^. A subset of the datasets were used in this analysis as we were primarily interested in the placing of the Irish Travellers within the context of Britain and Ireland, not the full structure found within Britain and Ireland. The results are presented in Fig. 1 in the form of a principal component analysis of fineStructure’s haplotype-based co-ancestry matrix (1A) and a dendrogram of the fineStructure clusters (1B).

We observe that 31 of 34 of the Irish Travellers cluster on the Irish branch, indicating a strong affinity with an Irish population ancestral to the current day “Traveller” and “settled” populations (Fig. 1B). One “Irish Traveller” is found within the *Borders 1* cluster, and two are found within the *Borders 2* cluster. These three individuals report full, or partial, English gypsie ancestry, a distinct and separate travelling population in Britain. One individual is found within the *Ireland 1* cluster, and two are found within the *Ireland 2* cluster. Traveller individuals within the *Ireland 2* cluster report recent settled ancestry, and we have no such genealogical data on the individual grouped within the *Ireland 1* cluster. Given their mixed ancestry, these individuals were excluded from subsequent F_st_, f_3_, and divergence estimate work.

The remaining 28 Irish Travellers in the fineStructure analysis were arranged into four clusters. These clusters were grouped on two separate branches (Fig. 1B), with *Traveller 1* (n = 7) and *Traveller 2* (n = 5) on the same branch, and *Traveller 3* (n = 5) and *Traveller 4* (n = 11) on a separate branch. The branch with clusters *Traveller 3* and *4*, forms an outgroup to the rest of the settled Irish and Irish Traveller clusters. These two branches of Irish Traveller clusters align closely with the split of Irish Travellers observed through PCA (Fig. S1). All the individuals who separate on the first principal component (henceforth “PCA group B”) are found in clusters *Traveller 3* and *4* (Fig. S2A), and nearly all the individuals who remain grouped with the settled Irish on principle component 1 (henceforth “PCA group A”) are found in clusters *Traveller 1* and *2* (Fig. S2A). The remaining PCA group A individuals are those Irish Travellers found in the aforementioned settled Irish or British clusters. This pattern is also repeated in the PCA (Fig. 1A), where members of *Traveller 1* and *2* cluster with the settled Irish, where *Traveller 3* and *4* individuals cluster separately.

Having identified distinct genetic groups of Irish Travellers, we investigated the correlation with Irish Traveller sociolinguistic features, specifically Shelta dialect, and Rathkeale residence (Fig. S2B,C, respectively). The majority of the Gammon speakers were members of clusters *Traveller 1* and *2.* All of *Traveller 1* consisted of Gammon speakers. The majority of clusters Traveller *3* and *4* consisted of Cant speakers, where all but one individual, for whom language identity is unknown, of *Traveller 4* were Cant speakers. We found that only clusters *Traveller 1* and *2* contain any Rathkeale Travellers, where 4 out of 5 individuals in *Traveller 2* are Rathkeale Travellers.

**Figure 2:**
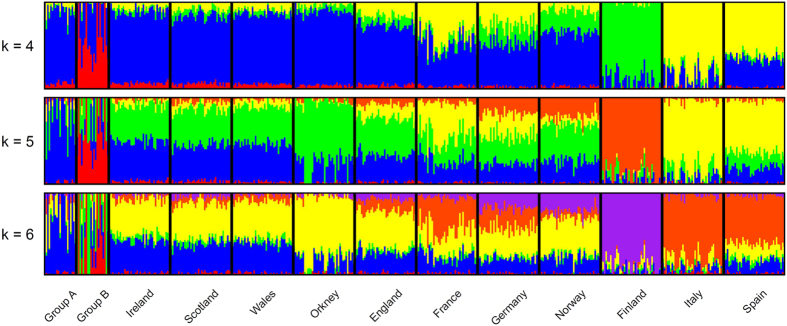
Ancestry profiles of the Irish Travellers, and neighbouring European populations by ADMIXTURE. Shown are the ancestry components per individual for the two groups of Irish Travellers (Group A and Group B), settled Irish, British, and European populations; modelling for 4 to 6 ancestral populations.

We next investigated population structure using the maximum-likelihood estimation of individual ancestries using ADMIXTURE (Figs 2 and S3). For this analysis we used a subset of the European Multiple Sclerosis dataset consisting of three northern European (Norway, Finland and Germany), two southern European (Italy and Spain), and a neighbouring population (France). We categorised the POBI British as English, Scottish, Welsh, and Orcadian. We further separated out the Irish Travellers to those in PCA group A and those in PCA group B.

At *k* = 4–6 (Fig. 2), we observe the well-described north-south divide in the European populations (*k* = 4), as well as Finland and Orkney (*k* = 5) differentiating due to their respective populations’ bottleneck and isolation. Although at lower values of *k* the Irish Travellers generally resemble the settled Irish profile (Fig. S3), at higher values of *k* two components are found to be enriched within the population. Each of these components is enriched in one of the two Irish Traveller PCA groups. Individuals with more than 20% of the “red” component when k = 5 belong to PCA group B and individuals with near 100% of “blue” component all belong to PCA group A (Fig. 2). The fact that even at *k* = 3 PCA group B gains its own ancestral component (Fig. S3) suggests strong group-specific genetic drift.

**Figure 3:**
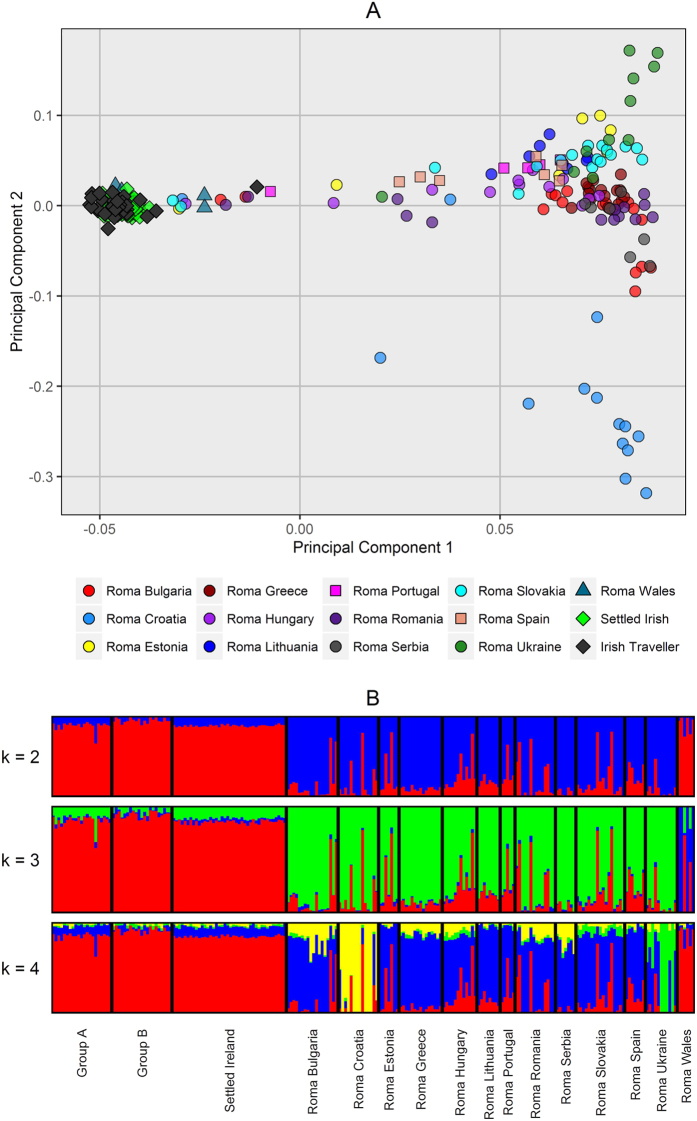
Comparison between the Irish Travellers, the settled Irish, and the European Roma. (**A**) The first and second components from principal component analysis using gcta64. (**B**) The ancestry profiles using ADMIXTURE, assuming 2 to 4 ancestral populations.

In order to investigate a possible Roma Gyspie origin of the Irish Travellers, we compared the Irish Travellers, and settled Irish to a dataset of Roma populations found within Europe^16^ using PCA and ADMIXTURE. The results broadly agree, with the Irish Travellers clustering with the settled Irish in the PCA plot, and resembling the settled Irish profile in ADMIXTURE analysis (see Fig. 3). There was no evidence for a recent ancestral component between the Irish Traveller and Roma populations. In addition, we formally tested evidence of admixture with f_3_ statistics in the form of f_3_(Irish Traveller; Settled Irish, Roma). We found no evidence of admixture either when considering all the Roma as one population, or in each individual Roma population’s case (all f3 estimates were positive).

Given the apparent structure between the Travellers and the settled Irish populations, we quantified genetic distance using F_st_ and “outgroup” f_3_ statistics. F_st_ analysis reveals a considerable genetic distance between the settled Irish and the Irish Traveller population (F_st_ = 0.0034, Table S1) which is comparable to values observed between German and Italian, or Scotland and Spain.

In order to further investigate sub-structure within the Irish Travellers, we performed F_st_ analysis on the Irish Traveller PCA (n = 2) and fineStructure (n = 4) groups, comparing them to the settled Irish (see also Table S1). The individuals belonging to cluster PCA group B are considerably more genetically distant from the settled Irish (F_st_ = 0.0086), relative to PCA group A (F_st_ = 0.0036). This could be explained by distinct founder events for PCA groups A and B, or that PCA group B has experienced greater genetic drift. The F_st_ estimates of the Irish Traveller clusters are higher than the PCA groups. The estimates of clusters *Traveller 1, 2*, and *3* range from 0.0052 to 0.0054. However, *Traveller 4* shows the highest F_st_ value (F_st_ = 0.0104), suggesting this cluster of individuals is responsible for the inflation of the PCA group B’s estimate. Generally, however, these results suggest that the general Irish Traveller population does not have a very recent source, i.e. within 5 generations or so. If we perform the same F_st_ analysis on two random groups of settled Irish see observe a F_st_ value < 1∙10^−5^.

To inform on whether lineage-specific drift is influencing the observed genetic distances between the Irish Travellers, the settled Irish and other neighbouring populations, we performed outgroup f_3_ analysis, using HGDP Yorubans as the outgroup. Such analysis can inform on whether PCA group B and *Traveller 4* do indeed represent an older Irish Traveller group, or a sub-group that has experienced more intense drift. When we compare PCA groups A/B to the settled Irish we see no significant difference between the two groups (see Table S2, A:settled f_3_ = 0.1694 (stderr = 0.0013), B:settled f_3_ = 0.1698 (stdrr = 0.0013), A:B f_3_ = 0.1700 (stderr = 0.0013)); with similar results for the fineStructure clusters (Table S2). These results suggest that PCA group B has experienced more drift than PCA group A, inflating the F_st_ statistic, which in turn has inflated the Irish Traveller population F_st_. We note however that f_3_ statistics may not be sensitive enough to detect differences from settled Irish to Traveller PCA groups A and B should the difference between A and B be a relatively limited number of generations.

### Divergence

A key question in the history of the Travellers is the period of time for which the population has been isolated from the settled Irish. In order to address this we utilized two methods, one based on linkage disequilibrium patterns and F_st_ (which we call T_F_), and one based on Identity-by-Descent (IBD) patterns (which we call T_IBD_).

The T_F_ method estimates the divergence to be 40 (±2 std.dev – obtained via bootstrapping) generations. Assuming an average generation time of 30 years the T_F_ method estimates that the divergence occurred 1200 (±60 – std.dev) years ago. The method also estimates the harmonic mean N_e_ for the two populations over the last 2000 years. The Irish Traveller estimate (1395, std.dev = 16 – obtained via bootstrapping) is considerably lower than the settled Irish estimate (6162, std.err = 122 – obtained via bootstrapping). However, the isolation of the Irish Travellers will artificially increase the F_st_ value and consequently inflate the T_F_ divergence estimate. We therefore estimated the divergence time with a different IBD-based method; as such an approach can accommodate genetic drift.

**Figure 4:**
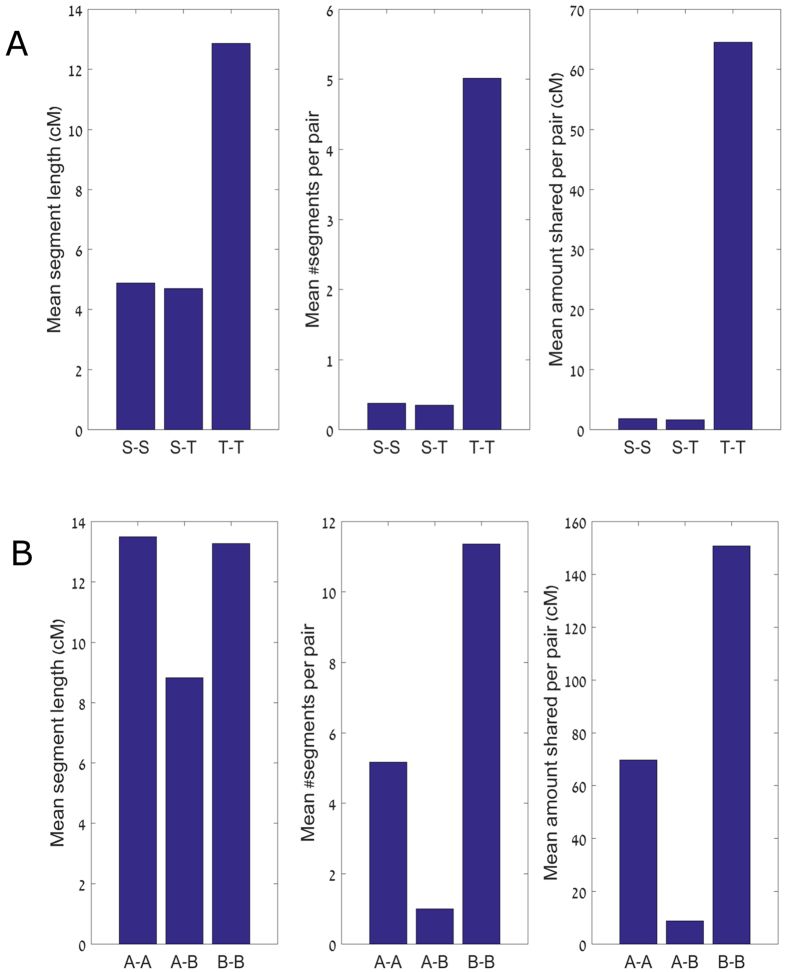
Extent of haplotype sharing between the settled Irish and the Irish Travellers, and between the two groups of Irish Travellers. (**A**) The number and lengths of shared segments within Settled Irish, within Traveller Irish, and between the groups. Left panel: The mean segment length; middle panel: the mean number of shared segments; right panel: the mean total sequence length (in cM) shared between each pair of individuals. (**B**) The number and lengths of shared segments within Traveller Group A, Traveller Group B, and between the groups. The format of the figure is as in (**A**).

We first identified IBD segment sharing within and between the Irish Travellers and our settled Irish subset. The Irish Travellers were found to share 35-fold more genetic material IBD (in cM per pair) than the settled population (Fig. 4A). Specifically, a pair of Travellers share, on average, 5.0 segments of mean length 12.9 cM, compared to 0.4 segments of mean length 4.9 cM for the settled population (Fig. 4A; segments with length >3 cM). Additionally we compared IBD sharing within and between the two PCA groups; A and B (Fig. 4B). We observe a greater amount of IBD segments shared within PCA group B than PCA group A. These sharing patterns are not due to familial sharing, as we have previously removed individuals with close kinship (see Supplementary Methods 1.3). Sharing between settled and Traveller Irish was of similar extent to that within the settled group (Fig. 4A), with no significant difference between the PCA groups A and B (p = 0.12, using permutations, for the difference in the number of segments shared with the settled) (Fig. S4). We used the number and lengths of segments shared within settled, within Travellers, and between the groups to estimate the demographic history of those populations, and in particular, the split time between these two groups.

**Figure 5:**
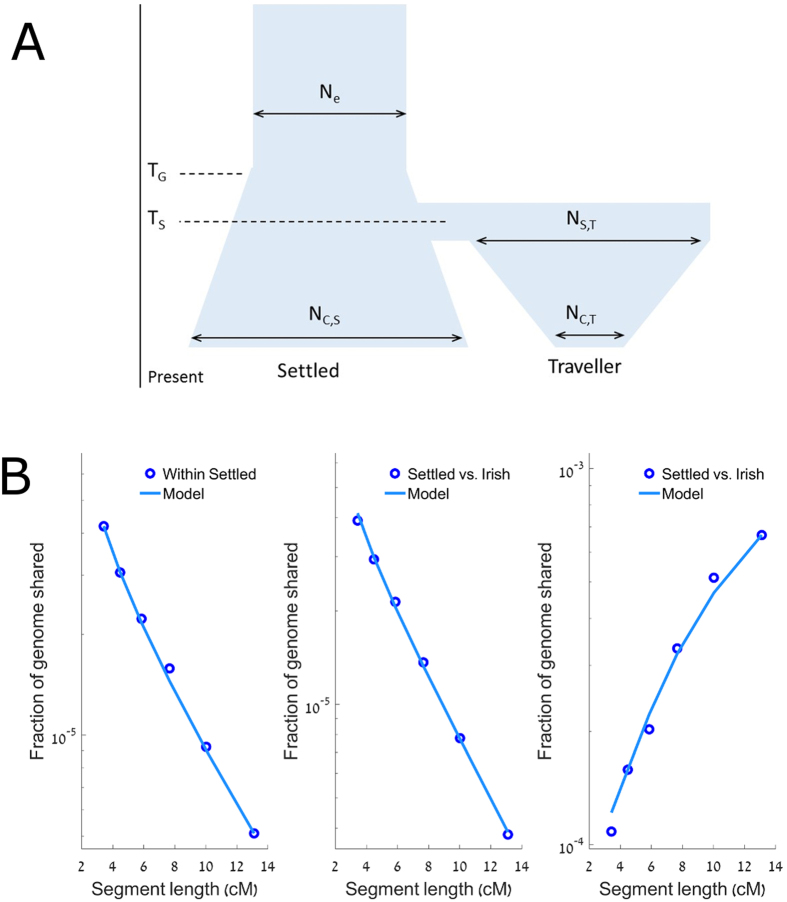
(**A**) The model used for demographic inference. The two populations were one ancestral population, with size N_e_, T_G_ generations ago. At this point the ancestral population started to grow exponentially until T_S_ generations ago, where the ancestral Traveller and settled populations split from each other, with N_S,T_ being the initial starting population size of the Traveller population. The settled population experienced continued exponential growth until the present, with a population size of N_C,S_. The Traveller population experienced a period of exponential contraction until the present, with a population of N_C,T_. (**B**) The proportion of the genome in IBD segments vs the IBD segments length. The total genome size and the sum of segment lengths were computed in cM. Left: sharing between pairs of settled Irish; middle: sharing between pairs of one settled and one Traveller individuals; right: sharing between pairs of Traveller Irish. Each data point is located at the harmonic mean of the boundaries of the length interval it represents.

Briefly, we used the method developed in Palamara *et al*.^17^ (see also Zidan *et al*.^18^). We assumed a demographic model for the two populations (Fig. 5A), in which an ancestral Irish population has entered a period of exponential expansion before the ancestors of the present day settled Irish and Irish Travellers split. After this split, the settled Irish continued the exponential expansion, whilst the Irish Travellers experienced an exponential population contraction. We then computed the expected proportion of the genome found in shared segments of different length intervals using the theory of ref. 17, and found the parameters of the demographic model that best fitted the data (see Supplementary Data 1.3, Fig. 5B, and Table 1).

The results of the model suggest the Irish Travellers and settled Irish separation occurred 12 generations ago (95% CI: 8–14). The results also support opposite trends in the effective population sizes (N_e_) of the settled and Traveller Irish since that split: while the settled population has expanded rapidly, the Irish Travellers have contracted (see Table 1). When restricting to the 12 members of PCA group A, the split time was estimated to be 15 generations ago (95% CI: 13–18) (Table 2). When restricting to the 16 members of PCA group B, the split time was 10 generations ago (95% CI: 3–14). We stress these results should be seen as the best fitting projection of the true history into a simplified demographic model, in particular given the limited sample sizes.

### Runs of Homozygosity

Consanguinity is common within the Irish Traveller population, and in this context we quantified the levels of homozygosity compared to settled Irish and world-wide populations^19^. We calculated the average total extent of homozygosity of each population using four categories of minimum length of Runs of Homozygosity (ROH) (1/5/10/16 Mb). Elevated ROH levels between 1 and 5 Mb are indicative of a historical smaller population size. Elevated ROH levels over 10 Mb, on the other hand, are reflective of more recent consanguinity in an individuals’ ancestry^10^. We also include average figures for the European Roma in the Irish Traveller – European analysis. Full European Roma ROH profiles are shown in Figure S5.

**Figure 6:**
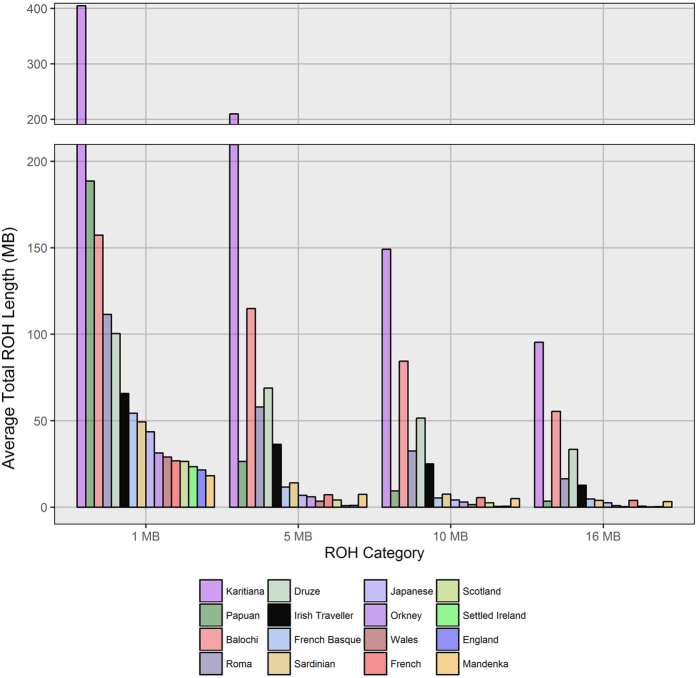
Extent of autozygosity in the Irish Travellers, settled Irish, select world-wide populations, and the European Roma. Shown, across four minimum lengths of runs of homozygosity (ROH), are the average lengths of ROH in each population. The average ROH burdens for the European Roma are the mean of means across the 13 Roma populations studied. These values are from a separate analysis, and collated with the wider European ROH values for reasons of SNP coverage between the different datasets.

As expected, the Irish Travellers present a significantly higher amount of homozygosity compared to the other outbred populations and to the European isolates the French Basque and Sardinian, which is sustained through to the larger cutoff categories of 10–16 Mb (see Fig. 6). Our results for the other world-wide populations agree with previous estimates^10^, with the Native American Karitiana showing the most autozygosity, and the Papuan population showing an excess of short ROHs. Two other consanguineous populations, the Balochi and Druze show slightly more homozygosity than the Irish Travellers, and the European Roma are most similar to the Travellers for both shorter and longer ROH.

These results indicate a higher level of background relatedness in the Irish Traveller population history. The high levels of ROH larger than 10 Mb in length reflect recent parental relatedness within the population. This is supported by the average F_ROH5_ in the Irish Travellers (F_ROH5_ = 0.015), which is slightly lower but comparable to the F_ROH5_ score found among Orcadian offspring of 1^st^/2^nd^ cousins (F_ROH5_ = 0.017)^20^.

Finally, in order to explore the potential of the Irish Traveller population for studying rare, functional variation for disease purposes, we tested minor allele frequency (MAF) differences between the settled Irish and the Irish Travellers from a common dataset of 560,256 common SNPs for 36 Traveller, and 2232 settled Irish individuals. We observed 24,670 SNPs with a MAF between 0.02–0.05 in the settled Irish population. We found that 3.29% of these SNPs had a MAF >0.1 in the Irish Traveller population. We tested the significance of this observation by calculating the same percentage, but taking a random 36 settled Irish sample instead of 36 Irish Travellers. We repeated this 1000 times and found no samples (p =< 0.001) with a greater percentage than 3.29 (mean = 1.3, std.dev = 0.11). This has additional implications for disease mapping within Ireland, as a proportion of the functional variants in the settled Irish population will be observed at a higher frequency in the Traveller population.

## Discussion

We have, using high-density genome-wide SNP data on 42 Irish Traveller individuals, investigated the genetic relationship between the Travellers and neighbouring populations and another nomadic European population, the Roma. For the first time we have estimated a time of divergence of the Irish Travellers from the general Irish population, and have also quantified the extent of autozygosity within the population.

We report that the Irish Traveller population has an ancestral Irish origin, closely resembling the wider Irish population in the context of other European cohorts. This is consistent with previous observations made using a limited number of classical markers^2^,^4^. In both our fineStructure and ADMIXTURE analyses, the Traveller population clusters predominantly with the settled Irish. Our fineStructure tree qualitatively agrees with the topology presented by Leslie *et al*.^21^, although there are some differences. For example, in the tree presented here, the Irish and individuals from south-west Scotland are grouped on one branch, with the rest of Scotland and England placed on a separate branch. fineStructure tree building is sensitive to the sample size, and due to the larger proportion of Irish genomes in our analysis, compared to Leslie *et al*.’s analysis (300 versus 44), it is not surprising that the Irish branch is placed differently.

We observe substructure within the Irish Traveller population, identifying (via fineStructure) four genetic clusters occupied only by Irish Travellers (Fig. 1B). These clusters align with the broad two way split in the Irish Traveller population we observe via allele frequency based PCA (Fig. S1). In addition, our fineStructure clusters reflect sociolinguistic affinities of the population, membership of the Rathkeale group (*Traveller* 2), and speakers of the Cant (*Traveller 4*) or Gammon (*Traveller1*) dialects of Shelta (Fig. S2). Our results, therefore, suggest that these groups represent genuine structure within the Irish Traveller population, rather than having by chance sampled broad family groups.

Several Irish Traveller individuals in the fineStructure analysis show an affinity either with British or settled Irish, demonstrating some genetic heterogeneity within the Irish Traveller population. This heterogeneity can be explained by recent settled ancestry or ancestry with other Travelling groups within Britain and Ireland. However, the existence of sole Irish Traveller genetic clusters suggest that there is some sub-structure within the population, and a larger follow up study is warranted to elucidate the extent of this structure, and the representative nature of the observed clusters.

It appears that the Traveller population has experienced lineage-specific drift, as demonstrated by the discordant F_st_ and f_3_ estimates between the Travellers and the settled Irish. F_st_ estimates of Traveller to Settled Irish genetic distance are comparable to that we observed between the Ireland and Spain (Table S1). However, when we estimate using f_3_ statistics (which is less sensitive to lineage-specific drift) the genetic distance, is reduced, and comparable to that observed between Irish and Scots. The theory of lineage-specific drift is also supported by the IBD analysis, which demonstrates very high levels of haplotype sharing within the Traveller population. Indeed, much of the overall genetic differentiation of the Travellers from the settled Irish is driven by the high F_st_ distance between the Irish Traveller PCA group B (specifically the *Traveller 4* cluster), and the settled Irish. This suggests that some subgroups within the Irish Travellers may have experienced greater genetic drift than others.

The dating of the origin of the Irish Travellers is of considerable interest, but this is distinct from the origins of each population. We have estimated the point of divergence between the Traveller and the settled Irish population using two different methods. Our LD-based (T_F_) method estimates a split 40 (±2 std.err) generations ago, or 1200 (±60 – std.err) years ago (assuming a generation time of 30 years). Our IBD-based method (T_IBD_) estimates 12 (8–14) generations, or 360 (240–420) years ago. However both estimates suggest that the Irish Travellers split from the settled population at least 200 years ago. The Irish Great Famine (1845–1852) is often proposed as a/the source of the Irish Traveller population, but results presented here are not supportive of this particular interpretation. The T_IBD_ method suggested differences between the PCA groups; whilst PCA group A seems to have split relatively early and remained relatively large, PCA group B seems to have split off more recently and quickly decline in size (Table 2). This might explain the higher degrees of genetic differentiation we see in PCA group B in our F_st_ and f_3_ analyses.

An important limitation of our dating analysis is that both the T_IBD_ and T_F_ approaches assume a single origin source, but there may have been multiple founding events contributing to the population present today. Both methods are further limited in that they do not model for subsequent gene flow in to the population. We would also consider the T_F_ date to be inflated, given the lineage-specific drift we and others have illustrated in the Traveller population, and its corresponding impact on F_st_ calculation. In the case of the T_IBD_ method, the sample size of the Irish Traveller cohort was too small to infer more complex demographic models (e.g. post-split gene flow or multiple epochs of growth/contraction for each group), due to the risk of over-fitting. A larger dataset is required to explore the possibility of dating distinct events for the Traveller clusters our analysis has resolved.

One of the hypothesised sources of the Irish Travellers is that they are a hybrid population between the settled Irish and the Roma. The results of our ADMIXTURE analysis would not support such a hypothesis, with none of the self-identified Irish Travellers showing ancestry components specific to the Roma populations. We did however detect one individual showing a significant proportion of a Roma-specific ancestral component. This individual self-reported Gypsie ancestry, and did not cluster with the clusters of sole Irish Traveller membership.

We have presented the first population-based assessment of autozygosity within the Irish Traveller population. Compared to other cosmopolitan populations, we observe within the Irish Travellers an excess of ROH and IBD segments. The ROH profile of the Irish Travellers is comparable to other consanguineous populations such as the Balochi of Pakistan and Druze of the Levant. However, of the populations we tested for ROH, the Irish Travellers were most similar to the European Roma, who are also an endogamous nomadic community. This, and the F_ROH5_ statistic for the Irish Travellers, agrees with previous observations of endogamy within the Irish Travellers^7^,^8^. Our homozygosity results would account for the well-documented higher prevalence of recessive disease within the Irish Traveller community^11^,^13^,^22^. The levels of homozygosity have clear importance in the medical genetics of the Irish Traveller population and together with the drift of rarer variants to higher frequencies in the Irish Travellers may greatly aid in the identification of rarer variants contributing to the risk of common disease within Ireland^23^, both for the settled and the travelling populations.

In summary, we confirm an ancestral Irish origin for the Irish Traveller population, and describe for the first time the genetics of the population using high-density genome-wide genotype data. We observe substructure within the population, a high degree of homozygosity and evidence of the “jackpot effect” of otherwise rare variants drifting to higher frequencies, both of which are of interest to disease mapping and complex trait genetics in Ireland. Finally we provide important insight to the demographic history of the Irish Traveller population, where we have estimated a divergence time for the Irish Travellers from the settled Irish to be at least 8 generations ago.

## Materials and Methods

### Study Populations

We assembled five distinct datasets; the Irish Travellers (n = 50), the Irish Trinity Student Controls^14^ (n = 2232), the People of the British Isles dataset^15^ (n = 2039), a dataset of individuals with European ancestry^24^ (n = 5964), individuals with Roma ancestry^16^ (n = 143), and a dataset of world-wide populations^19^ (n = 931). For more details of each dataset, see Supplementary Data 1.1.

The Irish Traveller cohort and data presented here were analysed within the guidelines and regulations put forward by the Royal College of Surgeons in Ireland Research Committee, and approved by the same Committee (reference number REC 1069). A waive of informed consent was granted by this Committee under an amendment of the same ethics reference number.

### Quality Control of Genotype Data

Each of the five cohorts was individually processed through a number of quality control steps using the software PLINK 1.9^25^,^26^. Only autosomal SNPs were included in the analysis. Individuals or SNPs that had >5% missing genotypes, SNPs with a minor allele frequency (MAF) <2%, and SNPs failing the HWE at significance of <0.001 were discounted from further analysis. Identity-by-Descent (IBD) was calculated between all pairs of individuals in each of the five datasets using the—genome function in plink, and one individual from any pairs that showed 3^rd^ degree kinship or closer (a pihat score ≥0.09) was removed from further analysis. Amongst the Irish Traveller cohort eight cryptic pairings closer than second-degree cousins were found, leaving 42 individuals for further analysis.

Individuals included from the European ancestry dataset^24^ were genotyped as part of a study of multiple sclerosis (MS), which included cases. As the HLA region contains loci strongly associated with multiple sclerosis (MS)^24^, for any analyses that included the European individuals from this MS study we omitted SNPs from a 15 Mb region around the HLA gene region, starting at 22,915,594 to 37,945,593. In order to restrict the MS cohort to individuals of European ancestry, we conducted principal component analysis (PCA) with gcta64 (v1.24.1)^27^ and outliers from each of the MS populations were also removed. This left the final 5964 individuals included in the MS European Cohort.

### Population Structure

FineStructure^28^ analysis was carried out on a combined dataset of Irish Travellers, Trinity Student Irish, and POBI British. As fineStructure is more sensitive to relatedness, instead of the previously described IBD threshold we removed one from each pair with a pihat score >0.06. Additionally we removed SNPs that were either A/T or G/C. This left a combined dataset of 34 Irish Travellers, 300 randomly chosen Irish from the Trinity Student dataset, and 828 British from the POBI dataset. The POBI samples were selected as follows; 500 individuals were chosen from England, and all 131 from Wales, 101 from Scotland, and 96 from Orkney. In order for the English individuals to be as representative as possible of English clusters identified previously^21^, the 500 consisted of; 200 randomly chosen from Central/South England, 50 randomly chosen from each of Devon and Cornwall, and 200 randomly chosen from the north of England. This final combined dataset had a total coverage of 431,048 common SNPs. Further details of the fineStructure analysis pipeline and its parameters are described in Supplementary Data 1.2.

In order to compare to other population structure visualisation methods we also performed allele frequency-based PCA using the software gcta64 (v1.24.1)^27^. Detailed methods are provided in Supplementary Data 3. This was applied to the same dataset as the fineStructure analysis, with the exception that we first pruned the dataset with regards to LD using plink 1.9^25^,^26^ with the—indep-pairwise command, using a window of 1000 SNPs moving every 50 SNPs, with an r^2^ threshold of 0.2. We also removed common SNPs that were either A/T or G/C, leaving 75,214 common SNPs.

Maximum likelihood estimation of individual ancestries was carried out using ADMIXTURE version 1.23^29^ and a dataset that had been pruned with respect to LD, as recommended by the authors^29^. This was achieved using plink 1.9^25^,^26^ with the—indep-pairwise command, using a window of 1000 SNPs moving every 50 SNPs, with an r^2^ threshold of 0.2. For this analysis we used a combined dataset of 42 Irish Travellers, 40 randomly selected Irish individuals from the Trinity Irish cohort, 160 individuals from the POBI dataset (40 randomly chosen English, Welsh, Orcadian, and Scottish individuals), and 40 random individuals from each of the following populations within the MS European dataset; France, Germany, Italy, Norway, Finland, and Spain. The combined dataset consisted of 83,759 SNPs (after the removal of A/T or G/C variants), and 476 individuals.

ADMIXTURE analysis was carried out on *k* = 2–7 populations, with 50 iterations of each *k* value. The iteration with the highest log-likelihood and lowest cross validation score was used for further analysis.

Inter-population fixation indexes between the populations were studied using the Weir and Cockerham method^30^ and the combined dataset used in ADMIXTURE analysis. The dataset was pruned with respect to LD using the same parameters as described above, leaving 83,759 common SNPs.

Due to the suspected lineage-specific drift in the Irish Traveller population history, we additionally calculated genetic distance using “outgroup” f_3_-statistics^31^, an extension of the f-statistics framework^32^. f_3_ is proportional to the shared genetic drift between two test populations and an outgroup population, and should therefore be less sensitive to the Irish Travellers lineage-specific drift than the F_st_ statistic. We performed this analysis on the same combined dataset used in F_st_ analysis, with the additional inclusion of 21 Yorubans from the HGDP dataset in order to act as an outgroup to the pair-wise comparisons. The combined dataset consisted of 245,594 common SNPs (after the removal of A/T or G/C variants). The outgroup f_3_ statistic was calculated using the software within the admixtools package^32^ using default settings.

### Divergence

In order to estimate a time of divergence between the Irish Travellers and the settled Irish we utilised two methods. The first, the T_F_ method, is based on a method first described by McEvoy *et al*.^33^ and uses linkage disequilibrium patterns between markers in discrete bins of recombination distances, and genetic distance measured by F_st_ in order to estimate a divergence time. The second, the T_IBD_ method, uses the sharing of Identical by Descent (IBD) segments and demographic modelling using this sharing data to estimate a time of divergence and is based on the methodology previously described in Palamara *et al*.^17^ and applied in Zidan *et al*.^18^. For more details of both methods, see Supplementary Data 1.3.

### Runs of Homozygosity Analysis

ROH analysis was carried out on a merged dataset of all individuals within the Irish Traveller, Trinity Student, and POBI cohorts, and a subset of the populations found within the Human Genome Diversity Project (HGDP) dataset. The HGDP populations were chosen to be i) representative of world-wide diversity of autozygosity, and ii) to compare the levels of autozygosity of the Irish Travellers to known endogamous populations such as the Balochi and Karitiana. The combined dataset had an overlap of 193,508 common markers.

With the exception of one parameter (the gap between consecutive SNPs, see below), we followed McQuillan *et al*.’s methodology^20^ for the ROH analysis; the window was defined as 1000 kb, moving every 50 SNPs, with 1 heterozygous position allowed and 5 missing positions allowed within the window. The run of homozygosity call criteria were defined as; 1/5/10/16 Mb minimum in length, 100 SNPs minimum within the window, the minimum marker density greater than 50 Kb/SNP. Due to the reduced SNP coverage in this dataset compared to previous analyses^10^,^20^ the largest gap between consecutive SNPs before ending a run of homozygosity call was changed to 500 Kb. We calculated F_ROH5_ as it had previously been shown to strongly correlate with the inbreeding coefficient F_PED_^20^. F_ROH5_ was estimated for the 17 populations, as per the equation below.


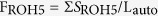


where S_ROH5_ is the total length of ROH found in an individual where runs are >5 Mb and L_auto_ is the total length of the autosomal genome (called as 2,673,768 kb here). The F_ROH5_ was averaged across the individuals to find the population mean of F_ROH5_.

### Relationship to European Roma

We performed several analyses in order to investigate the relationship between Irish Travellers and European Roma. Firstly, we assembled a merged dataset that included the full Irish Traveller, Trinity Student, and European Roma datasets. We additionally removed any variants that were A/T or G/C. For subsequent PCA and ADMIXTURE analysis the combined Roma dataset was pruned for LD, using a window of 1000 SNPs, moving every 50 SNPs with a r^2^ inclusion threshold of 0.2 in PLINK, leaving 66,099 common SNPs.

Secondly, PCA was performed using gcta64 v1.24.1^27^, creating a genetic relationship matrix, and then generating the first 10 principal components. Thirdly we applied ADMIXTURE on a reduced combined dataset that included all Irish Traveller and European Roma individuals, but only 40 of the Trinity Student Irish. ADMIXTURE was used with the same parameters as above, modelling for 2–4 ancestral populations. Finally, we compared the levels of homozygosity between the Irish Travellers, Trinity Student Irish, and European Roma - using the full combined Roma dataset, with 148,362 common SNPs and using the parameters described above.

Thirdly, we formally tested evidence for admixture using admixture f_3_ statistics^32^ in the form f_3_ (Traveller; Settled, Roma) using the full Trinity Irish dataset, a reduced European Roma dataset excluding the Welsh Roma (due to their outlier status in the rest of the dataset^16^), and a reduced dataset of Irish Travellers belonging to Irish Traveller clusters identified in fineStructure analysis (see Results). This combined dataset consisted of 148,914 SNPs.

## Additional Information

**How to cite this article:** Gilbert, E. *et al*. Genomic insights into the population structure and history of the Irish Travellers. *Sci. Rep.* **7**, 42187; doi: 10.1038/srep42187 (2017).

**Publisher's note:** Springer Nature remains neutral with regard to jurisdictional claims in published maps and institutional affiliations.

## Supplementary Material

Supplementary Data

## Figures and Tables

**Table 1:** The best fitting parameters for the T_IBD_ model, with the 95% confidence intervals (CI) shown below.

Parameter	N_A_	N_S,T_	N_C,S_	N_C,T_	T_S_	T_G_
Best fit	89,000	600,000	4.7∙10^6^	19	12	14
95% CI	84,000–96,000	1.7∙10^4^–3.0∙10^7^	8.8∙10^5^–8.6∙10^9^	7–45	8–14	12–19

Shown are the Irish ancestral effective population size (N_A_), the initial Traveller effective population size (N_S,T_), the current Irish (N_C,S_) and Traveller (N_C,T_) effective population sizes, and the time in generations of the split (T_S_) and start of exponential Irish growth (T_G_).

**Table 2:** The best fitting parameters for the T_IBD_ model, with the 95% confidence intervals (CI) shown below, considering only individuals from the PCA groups A or B.

Parameter	PCA Group	N_A_	N_S,T_	N_C,S_	N_C,T_	T_S_	T_G_
Best fit	A	86,000	21,000	7.6∙10^6^	450	15	15
95% CI	A	81,000–97,000	2700–3.3∙10^5^	1.1∙10^6^–4.5∙10^9^	100–6500	13–18	11–18
Best fit	B	90,000	56,000	1.5∙10^8^	8	10	13
95% CI	B	84,000–96,000	30–5.4∙10^6^	2.2∙10^6^–2.5∙10^10^	3–700	3–14	12–16

Shown are the Irish ancestral effective population size (N_A_), the initial Traveller effective population size (N_S,T_), the current Irish (N_C,S_) and Traveller (N_C,T_) effective population sizes, and the time in generations of the split (T_S_) and start of exponential Irish growth (T_G_).
