# Supplementary material for: Genomic insights into the population structure and history of the Irish Travellers
Source: Sci Rep. 2017 Feb 9;7:42187. doi: 10.1038/srep42187 (PMC5299991; doi:10.1038/srep42187)
Supplement: Supplementary Data [file srep42187-s1.pdf]

Title

Genomic insights into the population structure and history of the Irish Travellers.

Authors

Edmund Gilbert[1], Shai Carmi[2], Sean Ennis[3], James F. Wilson[4,5¶], Gianpiero L. Cavalleri[1¶*]

Supplementary Data

Title 1
Authors 1
Supplementary Data 1
Supplementary Data 1 –Methods 2
Supplementary Data 1.1 – Study Populations 2
Supplementary Data 1.2 – fineStructure Analysis 3
Supplementary Data 1.3 – Divergence Methods 5
Supplementary Data 2 - Irish Traveller Surnames 9
Supplementary Data 3 - Principal Component Analysis 12
Figure S1 – Principle Component Analysis of Irish Travellers, and Irish and British Individuals 13
Figure S2 – Irish Traveller fineStructure Cluster Demographics 14
Figure S3 –ADMIXTURE Graphs 15
Table S1 - F_{st} Statistics 16
Table S2 – Outgroup f_3 Statistic 17
Figure S4 – IBD Sharing Between Irish Traveller PCA Groups 18
Figure S5 – European Roma Runs of Homozygosity 19
Figure S6 – Comparison of Autozygosity and Ancestry Proportions of Traveller Groups A and B 20
References 22

Supplementary Data 1 –Methods

Supplementary Data 1.1 – Study Populations

We assembled five distinct datasets in our analysis of the Irish Travellers:

Irish Travellers Cohort (N = 50): This cohort was assembled using the following criteria; participants must have at least three grandparents with surnames traditionally associated with the Traveller population, only one member per surname was recruited, recruitment was spread evenly across the four provinces of Ireland. Recruitment also included four individuals with full or partial English Gypsie ancestry, for comparative purposes. Due to the nomadic nature of the Traveller community it is difficult to control for geographic representation, but during recruitment every effort was made to obtain a true representation of the island-wide Irish Traveller genetic diversity (see Supplementary Data 2 for further details). Information on Cant and Gammon speakers was provided by Travellers Michael and Nell McDonagh (*pers. comm*).

Saliva samples were collected using Oragene OG-250 (DNAGenotek, Canada) collection devices, and DNA extracted according to standard protocol. The Irish Traveller samples were genotyped on an Illumina OmniExpress 24 system at the Wellcome Trust Clinical Research Facility, Western General Hospital, Edinburgh, according to manufacturer's instructions.

The Irish Traveller cohort and data presented here was analysed within the guidelines and regulations put forward by the Royal College of Surgeons in Ireland Research Committee, and approved by the same Committee (reference number REC 1069). A waive of informed consent was granted by this Committee under an amendment of the same ethics reference number.

Trinity Student Study Cohort (N = 2,232): This cohort consisted of 2232 students recruited from Trinity College Dublin [1]. The sample was included as a representation of the settled Irish population. Genotype information was generated using the Illumina 1M HumanOmni1-Quad chip.

People of the British Isles Cohort (N = 2,039): This cohort consisted of British individuals from the WTCCC2 People of the British Isles (POBI) Study[2]. Genotype information from Illumina 1.2M platform was accessed via EBI, accession number EGAD00010000632.

Multiple Sclerosis (MS) European Cohort (N = 5,964): In order to provide wider European context, we included European individuals from the WTCCC2 Multiple Sclerosis (MS) Study[3]. Genotype information from Illumina Human660-Quad chip platform was accessed via EGA, accession number EGAD00000000120.

European Roma Cohort (N = 143): This sample included 143 individuals from Roma populations across Europe[4]. The samples were previously genotyped on the Affymetrix 6.0 platform[4].

Human Genome Diversity Project (HGDP) Dataset (N = 931): Individuals from this dataset [5] were previously genotyped on the Illumina HumanHap650K platform[5].

Supplementary Data 1.2 – fineStructure Analysis

fineStructure[6] was used to investigate population structure within the Irish Traveller population, on a dataset including 34 Irish Travellers, 300 randomly chosen Irish from the Trinity Student dataset[1], and 828 British from the People of the British Isles (POBI) dataset[2]. This sample size from the POBI study cohort is due to the coverage of specific regions around the British Isles found with the POBI dataset. 500 individuals were chosen from England, and all 131 from Wales, 101 from Scotland, and 96 from Orkney were also included. The sample of Irish Travellers was reduced because of the more stringent Identity by Descent (IBD) threshold needed for fineStructure analysis. As a result we removed one individual from each pair with a pihat score (obtained by PLINK[7, 8] with the --genome function) > 0.05.

The final dataset of 1,162 individuals and 431,048 common SNPs was phased using SHAPEIT[9] v2.r790, and the resultant hap files were converted to Chromopainter format using the

“impute2chromopainter2.pl” script (downloaded at http://www.paintmychromosomes.com/). For the phasing and conversion we used genetic map build 37 downloaded with SHAPEIT.

fineStructure analysis was performed by the combined software fineStructure 2.0.8, which includes the software Chromopainter, Chromocombine, and fineStructure. Chromopainter uses linkage disequilibrium between SNPs (the ‘linked’ model) to generate a coancestry matrix between the recipient and donor individuals in the analysis, and records the average haplotype sharing between the two. This co-ancestry matrix records the expected number of “chunks” each individual copies from each donor individual/population. Chromocombine is a software designed to combine the data output from Chromopainter (which can analyse each chromosome separately) into a single final coancesty matrix. fineStructure performs Bayesian cluster modelling on this final matrix by a Markov Chain Monte Carlo (MCMC) algorithm, and then performs additional “tree building” steps to improve the MCMC clustering and to hierarchically order these clusters into a dendrogram.

Chromopainter was applied using default settings, with the exception of specifying the number of ‘chunks’ per region to 50 as other analyses[10] have found that British and Irish individuals share relatively longer haplotypes than average. We ‘painted’ each individual using every other individual in the analysis as a donor using the –a 0 0 switch. Principal component analysis (PCA) was performed on the coancestry matrix, in R using the scripts supplied with fineStructure (accessed from http://www.paintmychromosomes.com/). PCA results were visualized in R using the ggplots package.

We then performed fineStructure analysis on the resultant coancestry matrix, where each Markov Chain Monte Carlo (MCMC) iteration offered the number of clusters (and the individual membership of each cluster) and samples according to their posterior probability (under the fineStructure model). Our analysis ran with 2,000,000 burnin iterations, 2,000,000 sampling iterations and sampling every 80,000 iterations. This was run twice as independent MCMC runs. The two runs

were then compared as part of the computational pipeline to check agreement with cluster membership. With the check successful, the MCMC iteration with the highest posterior probability, we performed 100 000 additional hill climbing steps which identify any splits or merges that improve the posterior. This final inferred tree is what we report. The final tree was visualized using scripts accessed from http://www.paintmychromosomes.com/, as well as using the dendextend R package.

Supplementary Data 1.3 – Divergence Methods

To estimate the time of divergence, two different methods were used; one that used LD patterns and population differentiation measured by F_{st} (henceforth T_F), and another that utilised sharing of identical-by-descent segments (T_{IBD}).

The T_F method was modified from that described by McEvoy et al, 2011[11]. Firstly, on the Irish Travellers found within either of the *Traveller 1*, *2*, *3*, or *4* fineStructure clusters, and the full Trinity Student dataset we generated the r^2 value of pairs of SNPs that were between 0.005 and 0.25 cM apart from a list of 560,256 common SNPs (after the removal of A/T or G/C variants), in each population, and chromosome separately. This was performed with PLINK, using the --r2 command, comparing SNPs up to 10,000 Kb apart, and setting the r^2 inclusion threshold as 0. SNP pairs were then organized into 250 recombination distance bins. As experimental sampling introduces chance LD, all individual pairwise r^2 values were adjusted by the sample size: r^2 - (1/n) where n is the sample size[12, 13]. r^2 values that were < (1/n) were discounted from subsequent analysis. The average r^2 for each distance bin was then obtained.

The effective population size (N_e) could then be estimated for each bin as $Ne = 1/(4c)*[(1/r^2)-2]$ where c is the recombination distance in Morgans, using the human genome build 37[11]. Different recombination distances reflect different times in a population's history, therefore linkage disequilibrium patterns over shorter recombination distances reflecting patterns in the more distant past, and longer distances reflecting more recent events. Recombination distance can be used to

estimate this time in t generations where t = 1/(2c). Therefore each recombination bin represents the N_e at different time periods. To obtain the overall N_e for each population, the harmonic mean of these N_e was obtained, using recombination distance bins ≥0.1 cM (up to 500 generation ago) as we were primarily interested in recent population histories.

To estimate the divergence time T_F, F_{st} can be used in conjunction with the harmonic mean N_e. The divergence time in t generations is $t = 2*N_e*F_{st}$, and was calculated for each chromosome separately. F_{st} was calculated using the same protocol in Population Structure (in main Materials and Methods) separately for each chromosome. N_e was obtained by finding the average of the harmonic mean of each of the two populations, using recombination distances ≥0.1 cM. McEvoy et al studied recombination distances ≤0.1 cM, but as we are primarily interested in more recent demographic events we opted to investigate LD patterns ≥0.1 cM. To generate the standard deviation of our N_e and generation time estimates, we used a bootstrapping procedure randomly sampling the 22 chromosomes with replacement to generate a sample of 1000 estimates for each of the two N_e and generation time estimates. We report the standard deviation of these bootstrapping procedures. As a control, we randomly chose two groups of thirty settled Irish and performed the same method, finding no statistically significant time of divergence.

The T_{IBD} method was previously described in Palamara et al., 2012[14] and applied in Zidan et al, 2015[15]. Briefly, a combined dataset of 560,256 SNPs for 28 Irish Travellers and 300 random settled Irish from the Trinity Student dataset was created. We used a reduced Irish Traveller sample, removing individuals with an excess of homozygosity, and only including those Irish Travellers found in any of the *Traveller 1-4* fineStructure clusters. We phased this combined dataset using SHAPEIT[9], on human genome build 37. We detected identical-by-descent (IBD) segments using Germline[16], with parameters; -bits 50, -err_hom 1, -err_het 1, and length of at least 3cM. We filtered out segments using Haploscore[17], which computes the minimal number of phase switches and genotyping errors required

for each segment to be truly shared IBD. We used Haploscore cutoff of 2, and additionally removed all segments with $\geq$5% overlap with sequence gaps. Finally, after inspecting the number of segments overlapping each genomic region at 1Mb resolution, we removed segments from five regions with particularly high levels of sharing, likely due to either structural variation, selection, or other sources of noise. Specifically, we removed the HLA region on chr6 and a region on chr8:4-17Mb known to have excess IBD sharing[18, 19], the *LCT* region on chr2, a region on chr10:16-19Mb, and a region on chr22:19-24Mb.

In our model for the recent demographic history of settled and Traveller Irish (Figure 5A), the ancestral population size was N_A until T_G generations ago. At that point, the population started growing exponentially. Then, T_S generations ago, the ancestral population has split into settled Irish and Irish Travellers. The settled population continued growing at the same rate, reaching $N_{C,S}$ at the present. The Travellers started at size $N_{S,T}$, and exponentially contracted until reaching size $N_{C,T}$ at the present.

To infer the model parameters, we divided the space of IBD segment lengths into six intervals of exponentially increasing size between 3-15cM. For each length interval, we computed, empirically, the proportion of the genome in IBD segments in length within the interval. This quantity was calculated for segments shared between pairs of settled individuals, segments shared between one settled and one Traveller individuals, and segments shared between pairs of Traveller individuals. For each proposed demographic model, we used the analytical results of Palamara et al[14] to compute the expected proportion of the genome in IBD segments for each length interval. To find the best fitting demographic model, we defined the error as the sum of the absolute values of the log-ratio of the theoretical and observed data points, summed over the three sharing categories. We minimized the error using the Nelder-Mead algorithm (Matlab's fminsearch). (Times were rounded and negative population sizes were converted to zeros.) We started the algorithm at a random initial guess, and then ran it four more times, each time from the starting point at the previous best fit. We repeated the process 500 times, and

recorded the demographic model achieving the minimal error. To obtain the 95% confidence intervals, we used bootstrapping over the chromosomes (500 repeats). For each parameter, we report the inferred values in the 2.5th and 97.5th bootstrap percentiles. Note that when inferring the model's parameters, we did not impose positive or negative growth rates in either population; rather, the growth rates were inferred from the data. Similarly, we allowed T_S to be either larger or smaller than T_G.

Supplementary Data 2 - Irish Traveller Surnames

Recruitment for the Irish Traveller cohort was preferentially chosen based on surnames that have been traditionally associated with Irish Travellers. In order to define what constituted an "Irish Traveller" surname, several sources were used:

1. An Sloinnteoir Gaeilge ("Directory of Irish Surnames") - A directory of as many Irish versions of surnames as its compilers could find for the island of Ireland, ranging from the indigenous Ó Murchú (Murphy) and Ó Flaitheartaigh (O'Flaherty) to obvious imports like Mac Seoin (Jones), Mac Uait (Watts) and Mac Conchearca (York/e). It is eighty pages in total.
2. RCI List - A list of observed surnames from the Report of the Commission on Itinerancy (RCI) (1963), with frequencies, borne by more than nine families of the 1,198 covered by the census of the 'itinerants'. There are 35 such surnames, representing 847 families, or 70% of the Irish Traveller families enumerated.
3. Flynn List - A list compiled by Dr Michael Flynn of Mullingar in 2000, by requesting information from local authorities in every county of the Republic but Dublin (deliberately omitted as "it was considered that the majority of Travellers in that county were migrants, or descendants of migrants, who had come to Dublin since the end of the Second World War"). All but Galway replied, so every county of the Republic but the two with the highest Traveller populations is included. (Aileen l'Amie was the sole source of information on Northern Ireland.) The 132 surnames listed by Flynn include all thirty-five noted by the RCI.
4. Sampson List - published in the Journal of the Gypsy Lore Society in 1890. This gives thirty-three surnames by Irish Province, some with 'Shelta' equivalents, but no indication of source. Making allowances for spelling discrepancies, six are listed in the 1963 Report; nine are on the Flynn list; and twenty-eight are in the Sloinnteoir, leaving five that occur in no other listing of Irish surnames, national or Traveller.
5. Thompson UK List - Thompson's 1923 list is confined to Irish Traveller families then to be found in the English Midlands. Surnames from this list were removed from consideration.
6. Folklore List - In 1953 the Folklore Commission distributed its "tinker questionnaire", including a request for surnames, throughout the country. Responses varied enormously in depth and detail, and in frequency by region. Dublin, for example, is again bypassed, and the coverage of Ulster is relatively poor. As a result, this cannot be treated as exhaustive. It is, however, very valuable, listing a total of 128 surnames. Just over half of these – sixty five – are mentioned by only one respondent, while a further ten are mentioned two or more times, but only within a single county.
7. Mac Greine List - In 1933, an unnamed "old tinker woman of 70 years" gave Pádraig Mac Gréine a list of Traveller surnames by Irish County/Province. The list is heavily weighted in favour of the Provinces Connacht and Munster.

From these sources several levels of priority were formulated. Individuals with surnames in the priority 1 or 2 categories were targeted. Of these individuals, three of their grandparental surnames had to have come from any of the 1-6 categories. The categories are as follows:

1. Those on the RCI and Flynn Lists
2. Those not in the RCI List, but on both the Flynn and Folklore Lists
3. Those only on the Folklore List
4. Those only on the Flynn List
5. Those only on the Sampson List
6. Those only on the Mac Greine List

Surnames in category 1 included:

- (Mc)(K)Inearney
- (O')Dono(g)hue
- (O')Donovan
- (O')Driscoll
- (O')Mongan(s)
- [Mac]Cart(h)y
- Berry
- Cash(man)
- Coffey/Coffee
- Corcoran
- Delaney
- Do(c)herty
- Doran
- Doyle
- Hand
- Harty
- Joyce
- Lawrence
- Maughan/m
- Murphy
- Nevin
- O'Leary
- Quilligan
- Stokes
- (O')Brien
- (O')Connors(s)
- (O')Flynn
- (O')Reilley/Riley
- Cawley
- Collins
- McDonagh
- O'Donnell
- Power(s)
- Sheridan
- Ward(e)

Surnames in category 2 included:

- (O')Sullivan
- Clarke
- Dukes
- West
- Barrett
- Boyle
- Burke
- Casey
- Cooney
- Crumlish
- Dinnegan
- Dooley
- Dundon
- Faulkner/Fortner
- Foley
- Gallagher
- Gammel
- Gavin

- Greene
- Hannifin
- Hean(e)y
- Hegarty
- Hogan
- Hutchinson
- Keena(n)
- Kelly
- Lynch
- Maguire
- Malone
- Mannion
- McCann
- McGinley
- McGrory
- Myers/Mears
- Price
- Purcell
- Quinn
- R(h)attigan
- Ryan
- Sherlock
- Smith
- Sweeney
- Tierney

Supplementary Data 3 - Principal Component Analysis

In order to compare to fineStructure's[6] haplotype based analysis, we additionally performed allele frequency based principal component analysis (PCA) using the software gcta64 (v1.24.1)[20]. This was performed on the same dataset used in the fineStructure analysis. We additionally pruned the dataset with regards to LD using Plink 1.9[7, 8] with the --indep-pairwise command, using a window of 1000 SNPs moving every 50 SNPs, with an r2 threshold of 0.2; leaving 95,214 common SNPs.

Supplementary Figure 1 show the results of principal components 1 and 2 with individuals coloured according to geographic location/population of origin. Along the first and second principal components the main populations are differentiated. Along the first component a subset of the Irish Travellers are clearly differentiated from the rest who cluster with the rest of the Irish (and British) individuals. We name the group of Irish Travellers who group with the Irish "PCA group A", and the group of Irish Travellers who separate from the rest "PCA group B".

Figure S1 – Principle Component Analysis of Irish Travellers, and Irish and British Individuals

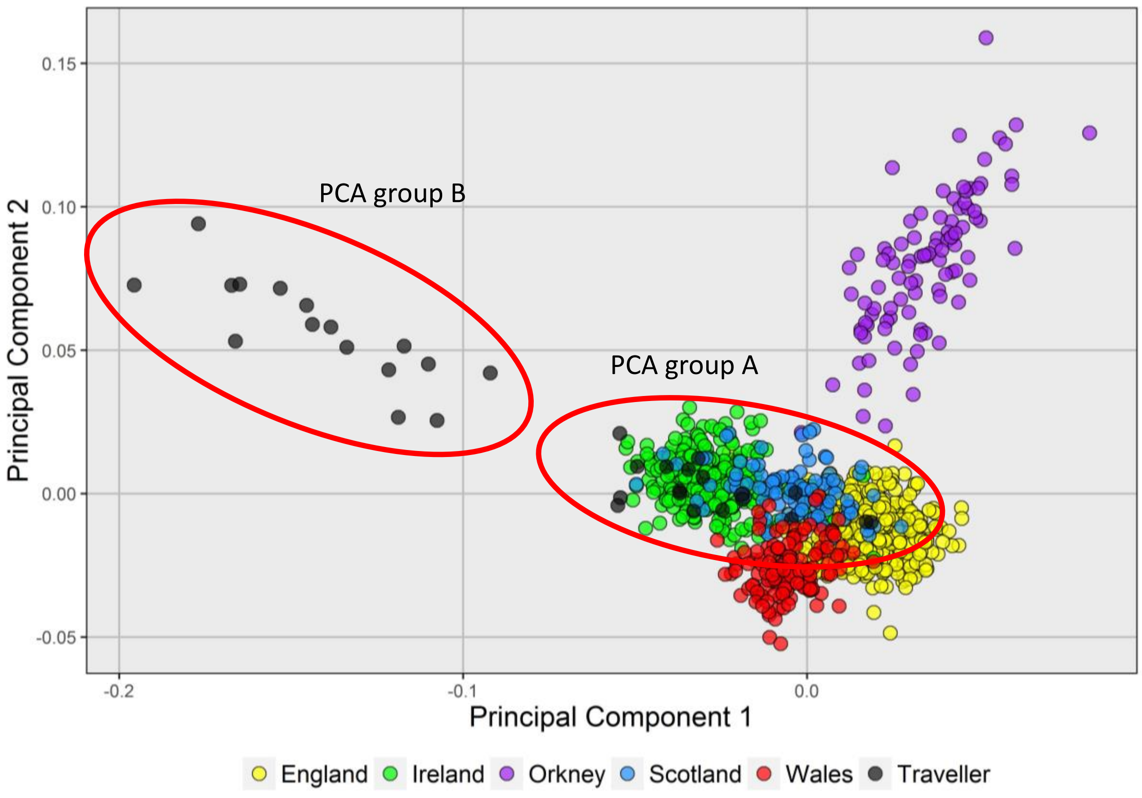

Figure S1 – Principal Component Analysis of the Irish Travellers with neighbouring Irish and British populations. Irish Traveller individuals are shown in black, and the two clusters of Irish Travellers are highlighted by red ellipses, which are labelled.

Figure S2 – Irish Traveller fineStructure Cluster Demographics

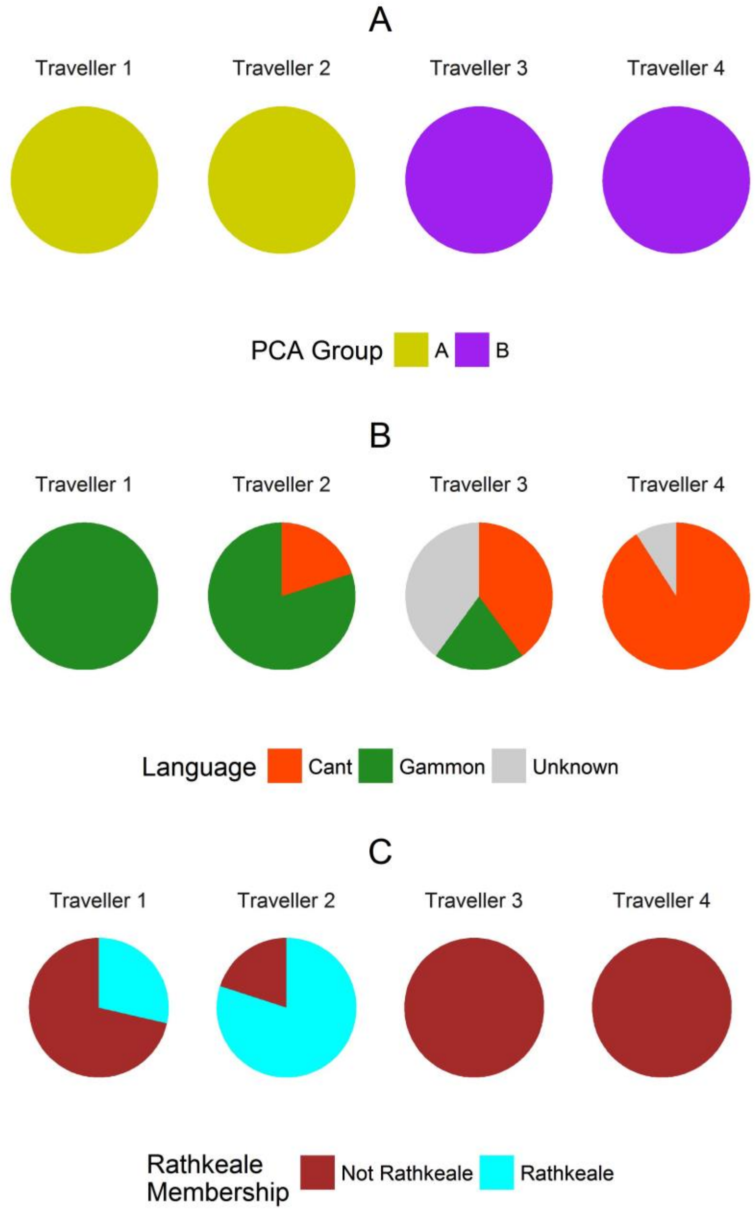

Figure S2 – The details of different demographics within the four fineStructure clusters of sole Irish Traveller membership. (A) The proportion of individuals in each cluster belonging to the groups identified in allele-frequency based principle component analysis. (B) The proportions in each cluster of individuals identified as speakers of any of the two different dialects of the Irish Traveller language, Shelta; Cant and Gammon. Also shown are the proportions of individuals where the language is unknown or not applicable. (C) The proportion of individuals that belong to the Rathkeale group of Irish Travellers in each cluster.

Figure S3 –ADMIXTURE Graphs

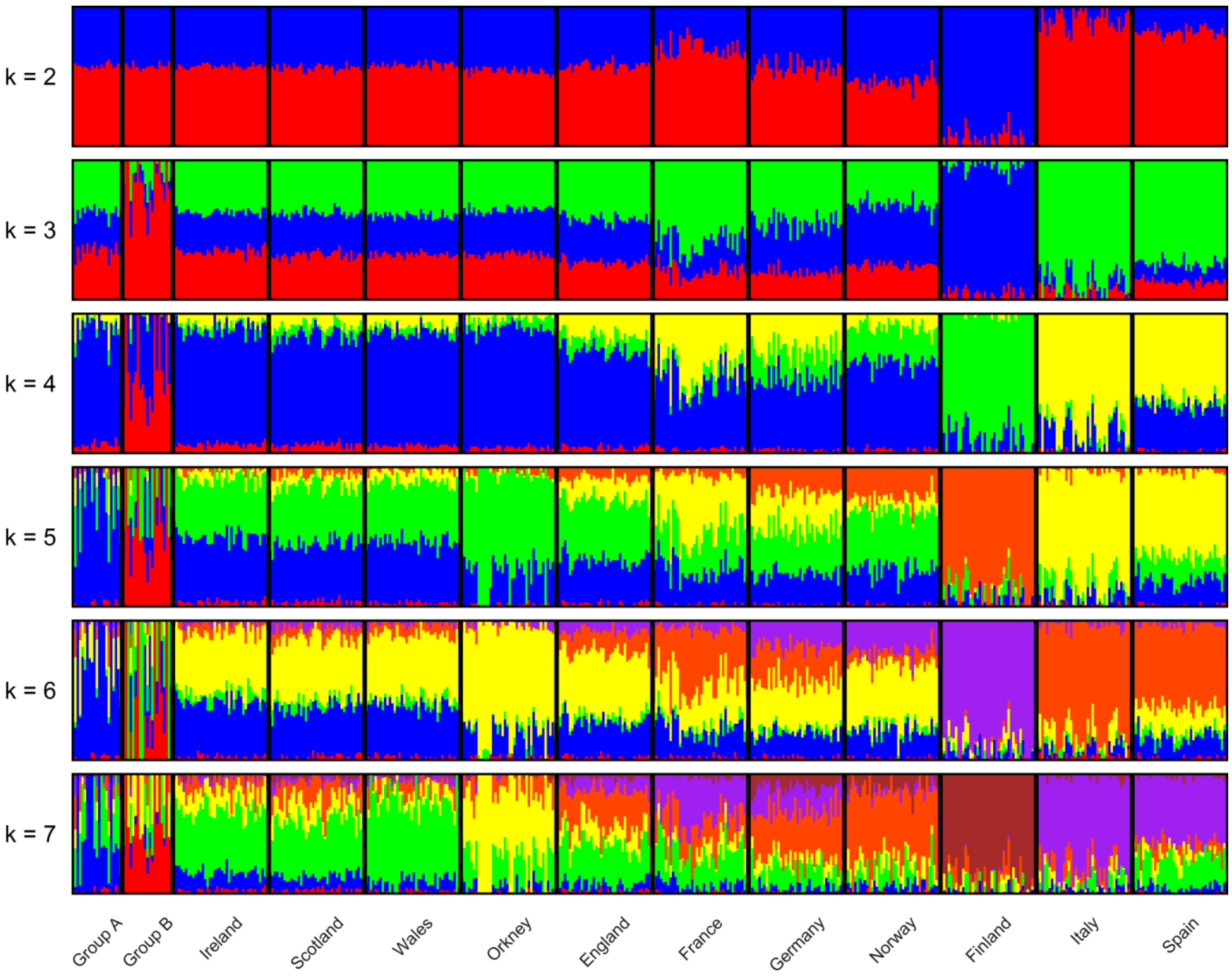

Figure S3 – The full ADMIXTURE[21] profiles for the Irish Travellers (separated into "Group A" and "Group B") and 11 other European populations, modelling for 2-7 ancestral populations.

Table S1 - F_{st} Statistics

Table S1 - F_{st} Comparisons of Irish Travellers with European Populations

Table of F_{st} estimates using the Weir and Cockerham method[22]. Shown are the different groups of Travellers; the PCA identified groups A and B (Trav A and B), and the fineStructure clusters (Trav 1, 2, 3, and 4). Our estimates of the other populations are in broad agreement with the values found in previous work[23, 24], and we observe that northern European populations are closer to each other than to southern European populations.

F_{st}	Traveller	Trav - A	Trav - B	Trav - 1	Trav - 2	Trav - 3	Trav - 4	Ireland	Scotland	Wales	Orkney	England	France	Germany	Norway	Finland	Italy	Spain
Traveller	-																	
Trav - A	-	-																
Trav - B	-	0.0101	-															
Trav - 1	-	-	-															
Trav - 2	-	-	-	0.0076	-													
Trav - 3	-	-	-	0.0085	0.0077	-												
Trav - 4	-	-	-	0.0149	0.0142	0.0023	-											
Ireland	0.0034	0.0036	0.0086	0.0054	0.0052	0.0053	0.0104	-										
Scotland	0.0028	0.0030	0.0079	0.0046	0.0049	0.0043	0.0098	0.0011	-									
Wales	0.0034	0.0039	0.0086	0.0057	0.0056	0.0053	0.0105	0.0016	0.0010	-								
Orkney	0.0042	0.0047	0.0094	0.0067	0.0063	0.0062	0.0113	0.0025	0.0017	0.0023	-							
England	0.0032	0.0037	0.0083	0.0054	0.0053	0.0051	0.0101	0.0014	0.0004	0.0009	0.0016	-						
France	0.0039	0.0042	0.0091	0.0058	0.0058	0.0058	0.0108	0.0022	0.0013	0.0017	0.0026	0.0008	-					
Germany	0.0041	0.0044	0.0093	0.0062	0.0059	0.0059	0.0111	0.0024	0.0012	0.0019	0.0024	0.0007	0.0007	-				
Norway	0.0042	0.0046	0.0095	0.0064	0.0061	0.0060	0.0114	0.0025	0.0015	0.0021	0.0022	0.0010	0.0017	0.0009	-			
Finland	0.0102	0.0107	0.0155	0.0126	0.0125	0.0123	0.0174	0.0086	0.0073	0.0081	0.0082	0.0069	0.0081	0.0060	0.0055	-		
Italy	0.0077	0.0079	0.0128	0.0095	0.0093	0.0098	0.0144	0.0061	0.0050	0.0053	0.0063	0.0041	0.0019	0.0035	0.0057	0.0124	-	
Spain	0.0058	0.0060	0.0108	0.0077	0.0073	0.0074	0.0125	0.0040	0.0031	0.0034	0.0044	0.0025	0.0006	0.0024	0.0038	0.0105	0.0013	-

Table S2 – Outgroup f_3 Statistic

Table S2 – Outgroup f_3 Comparisons of Irish Travellers with European Populations

The measures of genetic drift between Irish Travellers and 10 other European populations by “outgroup” f_3 statistics[25]. Shown are the different groups of Travellers; the PCA identified groups A and B (Trav A and B), and the fineStructure clusters (Trav 1, 2, 3, and 4). Yorubans from the HGDP dataset[5] were used as an outgroup. The lower triangle shows the f_3 values, and the upper triangle shows the standard error computed by the admixtools software[26].

F_3	Traveller	Trav A	Trav B	Trav 1	Trav 2	Trav 3	Trav 4	Ireland	Scotland	Wales	Orkney	England	France	Germany	Norway	Finland	Italy	Spain
Traveller	-	-	-	-	-	-	-	0.0013	0.0013	0.0013	0.0013	0.0013	0.0013	0.0013	0.0013	0.0013	0.0012	0.0012
Trav A	-	-	0.0013	-	-	-	-	0.0013	0.0013	0.0013	0.0013	0.0013	0.0013	0.0013	0.0013	0.0013	0.0012	0.0013
Trav B	-	0.1700	-	-	-	-	-	0.0013	0.0013	0.0013	0.0013	0.0013	0.0013	0.0013	0.0013	0.0013	0.0012	0.0012
Trav 1	-	-	-	-	0.0014	0.0014	0.0014	0.0013	0.0013	0.0013	0.0013	0.0013	0.0013	0.0013	0.0013	0.0013	0.0013	0.0013
Trav 2	-	-	-	0.1704	-	0.0013	0.0013	0.0013	0.0013	0.0013	0.0013	0.0013	0.0013	0.0013	0.0013	0.0013	0.0013	0.0013
Trav 3	-	-	-	0.1701	0.1710	-	0.0014	0.0013	0.0013	0.0013	0.0013	0.0013	0.0013	0.0013	0.0013	0.0013	0.0013	0.0013
Trav 4	-	-	-	0.1696	0.1701	0.1766	-	0.0013	0.0013	0.0013	0.0013	0.0013	0.0013	0.0013	0.0013	0.0013	0.0012	0.0013
Ireland	0.1695	0.1694	0.1698	0.1692	0.1698	0.1698	0.1697	-	0.0013	0.0013	0.0013	0.0013	0.0012	0.0012	0.0013	0.0013	0.0012	0.0012
Scotland	0.1693	0.1692	0.1696	0.1691	0.1694	0.1698	0.1695	0.1693	-	0.0013	0.0013	0.0013	0.0013	0.0013	0.0013	0.0013	0.0012	0.0012
Wales	0.1692	0.1690	0.1695	0.1688	0.1693	0.1697	0.1694	0.1694	0.1692	-	0.0013	0.0013	0.0013	0.0013	0.0013	0.0013	0.0012	0.0012
Orkney	0.1689	0.1688	0.1692	0.1685	0.1691	0.1694	0.1691	0.1691	0.1690	0.1689	-	0.0013	0.0013	0.0013	0.0013	0.0013	0.0012	0.0012
England	0.1683	0.1682	0.1686	0.1680	0.1685	0.1687	0.1686	0.1684	0.1685	0.1685	0.1684	-	0.0013	0.0013	0.0013	0.0013	0.0012	0.0012
France	0.1668	0.1667	0.1671	0.1666	0.1670	0.1672	0.1671	0.1669	0.1669	0.1670	0.1667	0.1665	-	0.0012	0.0013	0.0013	0.0012	0.0012
Germany	0.1676	0.1676	0.1679	0.1674	0.1678	0.1681	0.1679	0.1678	0.1679	0.1678	0.1678	0.1675	0.1661	-	0.0013	0.0013	0.0012	0.0012
Norway	0.1687	0.1687	0.1690	0.1684	0.1691	0.1692	0.1689	0.1688	0.1689	0.1689	0.1689	0.1685	0.1667	0.1681	-	0.0013	0.0012	0.0012
Finland	0.1664	0.1663	0.1667	0.1661	0.1666	0.1669	0.1666	0.1665	0.1668	0.1665	0.1667	0.1662	0.1642	0.1663	0.1677	-	0.0012	0.0012
Italy	0.1624	0.1623	0.1627	0.1622	0.1625	0.1625	0.1628	0.1625	0.1626	0.1627	0.1624	0.1624	0.1622	0.1622	0.1622	0.1595	-	0.0012
Spain	0.1637	0.1636	0.1640	0.1633	0.1640	0.1639	0.1640	0.1638	0.1638	0.1639	0.1636	0.1634	0.1632	0.1630	0.1634	0.1607	0.1604	-

Figure S4 – IBD Sharing Between Irish Traveller PCA Groups

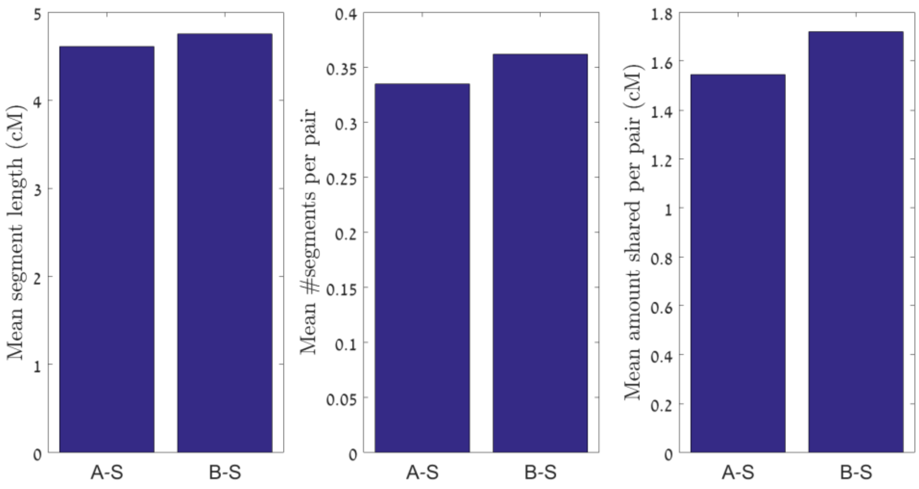

Figure S4 - The sharing of IBD segments between PCA group A and B, and the settled Irish. Left panel: The mean segment length shared between PCA group A and B, and the settled Irish. Middle panel: The mean number of IBD segments between PCA group A and B, and the settled Irish. Right panel: The mean total length of IBD segment sharing (cM) between PCA group A and B, and the settled Irish.

Figure S5 – European Roma Runs of Homozygosity

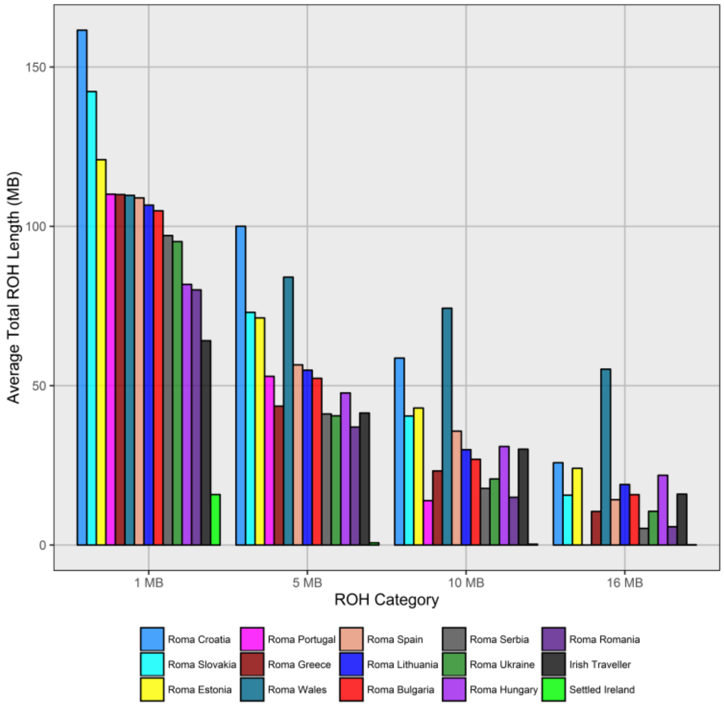

Figure S5 – The extent of homozygosity by Runs of Homozygosity (ROH) analysis in European Roma populations compared to the Irish Traveller and settled Irish populations. Shown is the population mean total length of ROH across four categories of minimum ROH length (1/5/10/16 MB).

Figure S6 – Comparison of Autozygosity and Ancestry Proportions of Traveller Groups A and B

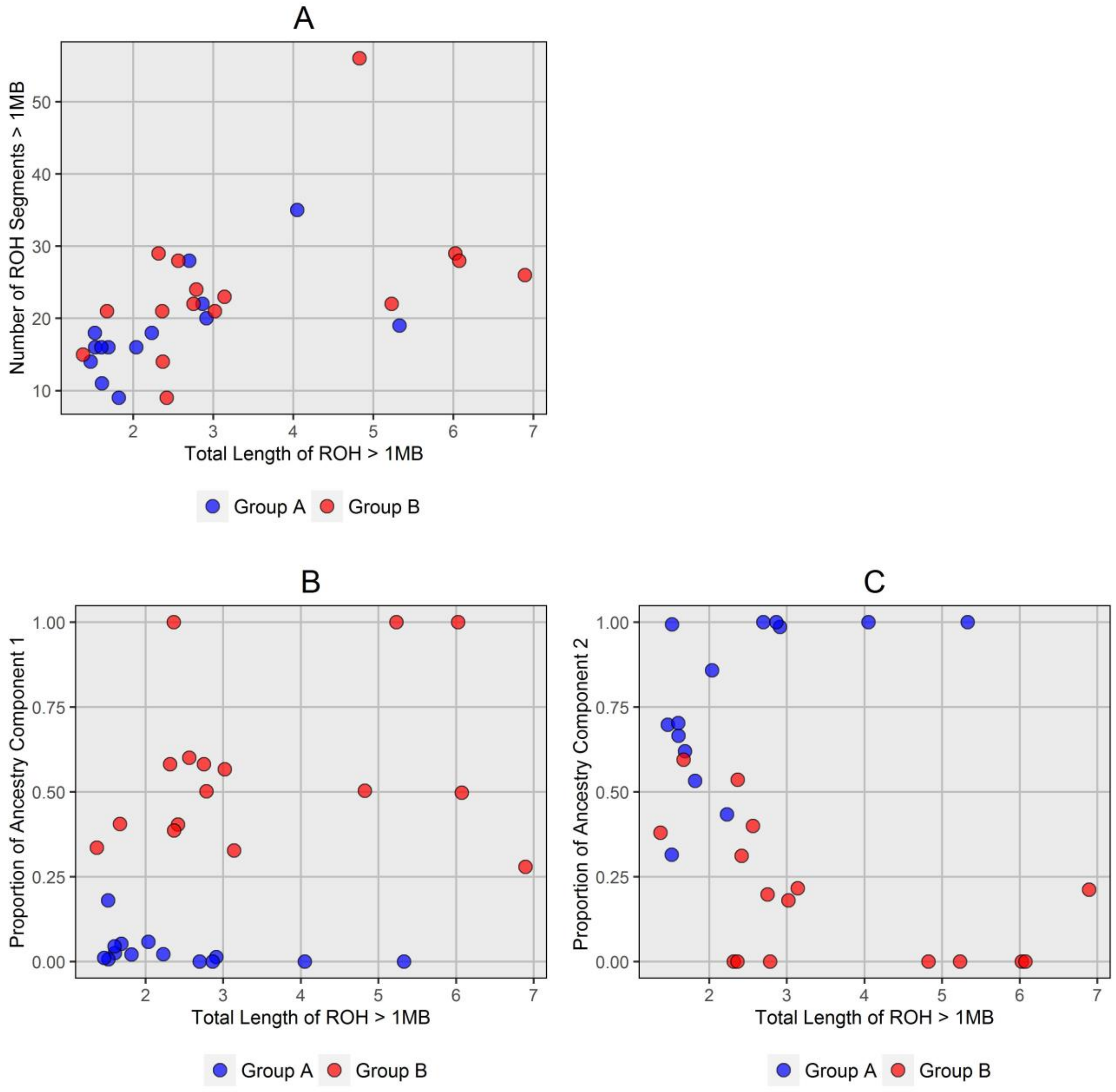

Figure S6 – (A) The total length of runs of homozygosity (ROH) > 1Mb versus the number of ROHs. (B) The total length of ROH > 1Mb versus the proportion of ancestral component 1 from ADMIXTURE analysis where *k* = 6 (the red component). (C) The total length of ROH > 1Mb versus the proportion of ancestral component 2 from ADMIXTURE analysis where *k* = 6 (the blue component). Each symbol is an individual Irish Traveller.

We tested the correlation between ancestry components 1 and 2 versus the levels of autozygosity in PCA groups B and A (respectively). We found a significant correlation between

autozygosity and ancestry component 2 in group A ($r2= 0.028$, $p= 0.030$), but the correlation between autozygosity and ancestral component 1 in PCA group B was insignificant ($r2= -0.020$, $p= 0.415$).

References

1. Desch, K., et al., *Linkage analysis identifies a locus for plasma von Willebrand factor undetected by genome-wide association.* Proc Natl Acad Sci USA, 2013. **110**(2): p. 588-93.
2. Winney, B., et al., *People of the British Isles: preliminary analysis of genotypes and surnames in a UK-control population.* Eur J Hum Genet, 2012. **20**(2): p. 203-10.
3. IMSGC and WTCCC2, *Genetic risk and a primary role for cell-mediated immune mechanisms in multiple sclerosis.* Nature, 2011. **476**(7359): p. 214-219.
4. Mendizabal, I., et al., *Reconstructing the Population History of European Romani form Genome-wide Data.* Curr Biol, 2012. **22**(24): p. 2342-9.
5. Li, J., et al., *Worldwide human relationships inferred from genome-wide patterns of variation.* Science, 2008. **319**(5866): p. 1100-4.
6. Lawson, D., et al., *Inference of population structure using dense haplotype data.* PLoS Genet, 2012. **8**(1): p. e1002453.
7. Purcell, S., et al., *PLINK: a tool set for whole-genome association and population-based linkage analyses.* Am J Hum Genet, 2007. **81**(3): p. 559-75.
8. Chang, C., et al., *Second-generation PLINK: rising to the challenge of larger and richer datasets.* Gigascience, 2015. **4**: p. 7.
9. Delaneau, O., J. Marchini, and J. Zagury, *A linear complexity phasing method for thousands of genomes.* Nature Methods, 2011. **9**(2): p. 179-81.
10. Leslie, S., et al., *The fine-scale population structure of the British population.* Nature, 2015. **519**: p. 309-14.
11. McEvoy, B., et al., *Human population dispersal "Out of Africa" estimated from linkage disequilibrium and allele frequencies of SNPs.* Genome Research, 2011. **21**(6): p. 821-9.
12. Weir, B. and W. Hill, *Effect of mating structure on variation in linkage disequilibrium.* Genetics, 1980. **95**(2): p. 477-88.
13. Tenesa, A., et al., *Recent human effective population size estimated from linkage disequilibrium.* Genome Res, 2007. **17**(4): p. 520-6.
14. Palamara, P., et al., *Length distributions of identity by descent reveal fine-scale demographic history.* Am J Hum Genet, 2012. **91**(5): p. 809-22.
15. Zidan, J., et al., *Genotyping of geographically diverse Druze trios reveals substructure and a recent bottleneck.* Eur J Hum Genet, 2015. **23**(8): p. 1093-9.
16. Gusev, A., et al., *Whole population, genome-wide mapping of hidden relatedness.* Genome Res, 2009. **19**(2): p. 318-36.
17. Durand, E., N. Eriksson, and C. McLean, *Reducing pervasive false-positive identical-by-descent segments detected by large-scale pedigree analysis.* Mol Biol Evol, 2014. **31**(8): p. 2212-22.
18. Gusev, A., et al., *The architecture of long-range haplotypes shared within and across populations.* Mol Biol Evol, 2012. **29**(2): p. 473-86.
19. Albrechtsen, A., I. Moltke, and R. Nielsen, *Natural selection and the distribution of identity-by-descent in the human genome.* Genetics, 2010. **186**(1): p. 295-308.
20. Yang, J., et al., *GCTA: a tool for genome-wide complex trait analysis.* Am J Hum Genet, 2011. **88**(1): p. 76-82.
21. Alexander DH, Novermbre J, and L. K, *Fast model-based estimation of ancestry in unrelated individuals.* Genome Research, 2009. **19**(9): p. 1655-1664.
22. Weir, B. and C. Cockerham, *Estimating F-statistics for the analysis of population structure. .* Evolution, 1984. **38**: p. 1358–70.
23. Heath, S., et al., *Investigation of the fine structure of European populations with applications to disease association studies.* Eur J Hum Genet, 2008. **16**(12): p. 1413-29.

24. Tian, C., et al., *European population genetic substructure: further definition of ancestry informative markers for distinguishing among diverse European ethnic groups.* Mol Med, 2009. **15**(11-12): p. 371-83.
25. Raghavan, M., et al., *Upper Palaeolithic Siberian genome reveals dual ancestry of Native Americans.* Nature, 2014. **505**(7481): p. 87-91.
26. Patterson, N., et al., *Ancient admixture in human history.* Genetics, 2012. **192**(3): p. 1065-93.
